# Isolation and Bioactive Characterization of *Berberis kaschgarica* Rupr-Derived Exosome-Like Nanovesicles: Exploring Therapeutic Potential in Atherosclerosis Pathogenesis

**DOI:** 10.3390/biology14060726

**Published:** 2025-06-19

**Authors:** Dilihuma Dilimulati, Nuerbiye Nueraihemaiti, Alhar Baishan, Sendaer Hailati, Alifeiye Aikebaier, Yipaerguli Paerhati, Wenting Zhou

**Affiliations:** 1 Department of Pharmacology, School of Pharmacy, Xinjiang Medical University, Urumqi 830017, China; dilihuma@stu.xjmu.edu.cn (D.D.); nurbiye@stu.xjmu.edu.cn (N.N.); alhar@stu.xjmu.edu.cn (A.B.); sendaer@stu.xjmu.edu.cn (S.H.); alifeiye@stu.xjmu.edu.cn (A.A.); yipaerguli@stu.xjmu.edu.cn (Y.P.); 2 Xinjiang Key Laboratory of Natural Medicines Active Components and Drug Release Technology, Urumqi 30017, China; 3 Xinjiang Key Laboratory of Biopharmaceuticals and Medical Devices, Urumqi 830017, China; 4 Engineering Research Center of Xinjiang and Central Asian Medicine Resources, Ministry of Education, Urumqi 830017, China

**Keywords:** *Berberis kaschgarica* Rupr.-derived exosome-like nanovesicles (BELNs), multi-omics, exosomes, miRNA, lipidomics, proteomics

## Abstract

*Berberis kaschgarica* Rupr.-derived exosome-like nanovesicles (BELNs), a type of plant-derived extracellular vesicle, consist of proteins, lipids, and nucleic acids. In this research, we employed differential centrifugation and ultracentrifugation techniques to isolate and purify BELNs. Subsequently, we conducted a comprehensive multi-omics analysis to systematically determine their physicochemical properties. Experiments were conducted in vitro with Human Umbilical Vein Endothelial Cells (HUVECs) to verify the therapeutic impact of BELNSs on atherosclerosis. The isolated BELNs exhibited a distinctive teacup-shaped exosome morphology. The extraction yield was approximately 2.1 × 10^13^ particles per milliliter, and the average particle size was measured to be 179.1 nm. These nanovesicles were lipid-rich. The protein content predominantly comprised cytoplasmic proteins. In-depth analysis revealed the presence of five highly conserved plant microRNAs: miR166, miR156, miR399, miR171, and miR395. These miRNAs are involved in regulating plant growth and responses to both biotic and abiotic stresses. Functional assays demonstrated that *Berberis kaschgarica* Rupr.-derived exosome-like nanovesicles substantially decreased the lipid deposition in HUVECs that was triggered by Palmitic Acid (PA). This research establishes the inaugural utilization of multi-omics platforms to systematically elucidate the bioactivity profile of BELNs from *Berberis kaschgarica* Rupr., thereby laying the groundwork for advancing its therapeutic potential.

## Simple Summary

Exosome-like nanovesicles are membrane-enclosed particles with round-shape morphology and signaling functions, which resemble mammalian cell-derived extracellular vesicles. In this study, we obtained exosomes from *Berberis kaschgarica* Rupr. and explored their therapeutic potential for atherosclerosis. Exosomes were isolated through ultracentrifugation following gradient purification, revealing an average particle size of 179.1 nm with an exosome-like morphology. These nanovesicles were lipid-rich. The protein content predominantly comprised cytoplasmic proteins. In-depth analysis revealed the presence of five highly conserved plant microRNAs: miR166, miR156, miR399, miR171, and miR395. These miRNAs are involved in regulating plant growth and responses to both biotic and abiotic stresses. Functional assays demonstrated that *Berberis kaschgarica* Rupr.-derived exosome-like nanovesicles substantially decreased the lipid deposition in Human Umbilical Vein Endothelial Cells that was triggered by Palmitic Acid. These findings suggest that *Berberis kaschgarica* Rupr. is a promising and abundant source of exosomes with potential applications in nanomedicine for therapeutic purposes.

## 1. Introduction

*Berberis kaschgarica* Rupr., a deciduous shrub (1.5–3 m tall) in Berberidaceae, is characterized by trigonous branching spines and elliptic obovate leaves. Distributed in arid/semi-arid northwest China (37–42° N, mainly in Tianshan southern foot alluvial fans and Tarim Basin western edge), its berry-like drupes are rich in alkaloids. Used in Uyghur medicine for liver/gallbladder diseases, as a natural food colorant, and valued for ecological restoration, the plant’s fruits serve as tea/dietary raw materials and traditional medicine for skin inflammation, hypertension, and other conditions, with reported effects including blood circulation promotion, stasis removal, choleresis improvement, hypertension prevention, and anti-inflammation [1]. Its roots, stems, and leaves, rich in alkaloids, also have significant medicinal value.

The isolation and functional profiling of exosomes derived from dietary plant sources have emerged as a rapidly evolving frontier in pharmaceutical and nutraceutical sciences. These naturally occurring vesicular structures demonstrate unique potential as biomolecule delivery platforms due to their capacity to encapsulate and transport bioactive phytochemicals with demonstrated therapeutic relevance. Exosomes, which feature a double-membrane structure, are carriers made up of lipids, proteins, and nucleic acids [2]. For millennia, botanical resources have constituted the pharmacopeic foundation across traditional medicinal systems, concurrently serving as progenitor materials for contemporary drug discovery. Emerging phytochemical investigations reveal conserved nano-architectures across diverse plant taxa—notably *Citrus limon* [3], *Vitis vinifera* [4], *Zingiber officinale* [5], and *Solanum lycopersicum* [6]—from which bioactive nanovesicles can be isolated through differential ultracentrifugation. These exosome-like nanovesicles (ELNs) [7] exhibit structural homology with mammalian counterparts, featuring a phospholipid bilayer membrane and characteristic saucer-shaped ultrastructure observable under cryo-electron microscopy. Previously, studies have focused on animal-derived ELNs. However, over the past decade, plant-derived exosome-like nanovesicles (PELNs) have gradually attracted the attention of researchers [8]. PELNS offer several advantages over animal ELNs, including wider availability, ease of large-scale production, reduced extraction costs, excellent biocompatibility, and reduced side effects [9]. Thus, they provide a sustainable and cost-effective therapeutic approach, thus overcoming the limitations of synthetic nanoplants.

Atherosclerosis (AS) is a chronic inflammatory disorder, initiated by endothelial dysfunction and structural alterations, encompassing the chronic inflammation of the vessel wall [10,11]. The atherosclerotic cardiovascular disease manifests predominantly through acute thrombotic events, notably myocardial infarction and cerebrovascular accidents, which collectively account for 31.8% of global mortality according to WHO 2023 data. Current clinical paradigms emphasize secondary prevention strategies targeting these terminal complications, as codified in recent American College of Cardiology/American Heart Association guidelines [12]. The atherosclerotic progression is driven by endothelial dysfunction, dyslipidemia, and prothrombotic hematological alterations. Hemodynamic shear stress activates endothelial mechanotransduction pathways, triggering NF-κB-mediated inflammatory cascades that enhance plaque vulnerability [13]. A key pathological feature is the oxidative modification of apolipoprotein B-containing lipoproteins (notably LDL), forming ox-LDL that accumulates in arteries via proteoglycan binding. This initiates foam cell formation and necrotic core development in unstable plaques [14,15].

To advance the study of *Berberis kaschgarica*-derived exosome-like nanovesicles (BELNs), we isolated BELNs and characterized them through multiple methodologies combined with omics-based analyses. This study lays the foundation for a more effective utilization of saffron flower waste, presenting novel prospects for its enhanced value in diverse therapeutic and biotechnological domains.

## 2. Materials and Methods

### 2.1. Materials

Instruments: Mini Refrigerated Centrifuge (Microfuge 20R, Beckman Coulter, Pasadena, CA, USA), Ultracentrifuge (CR22N, Hitachi, Tokyo, Japan), Transmission Electron Microscope (TEM, HT7800, Hitachi, Tokyo, Japan), Nanoparticle Tracking Analyzer (ZetaView S/N 24-1078, PARTICLE METRIX, Munich, Germany), Multimode Microplate Reader (Varioskan LUX, Thermo Scientific, Waltham, MA, USA), Nucleic Acid Quantitation System (Quantus, Promega, Madison, WI, USA), Thermal Cycler (PCR System, ABI 7500 Fast, Thermo Scientific, Waltham, MA, USA), Quantus Fluorometer (Navigator GM200, Promega, USA), and Automated Nucleic Acid/Protein Analysis System (Qsep 100, BIOptic, Jiangsu, China).

Materials: *Berberis kaschgarica* Rupr. (Kaschgarica, Akto, China), Isopropyl alcohol (A461-212), methyl alcohol (019393-M1), and a BCA kit (A55861) were obtained from Thermo Scientific (USA). Paraformaldehyde (Sinopharm Chemical Reagent Co., Shanghai, China), Glutaraldehyde (Guangzhou Chemical Reagent Factory, Guangzhou, China). LC/MS-grade water (1.15333.4000) and acetonitrile (1.00030.4008) were purchased from Merck (Darmstadt, Germany).

### 2.2. Isolation of BELNs from Berberis kaschgarica Rupr.

Fresh *Berberis kaschgarica* fruits were juiced and filtered. The clarified plant homogenate was aliquoted into 50 mL polycarbonate tubes (Beckman Coulter) for sequential differential centrifugation: Primary clarification: 1000× *g*, 4 °C, 30 min (Sorvall ST 40R, FIBERlite F15-8x50cy rotor); cellular debris removal: 2000× *g*, 4 °C, 30 min; macroparticle elimination: 10,000× *g*, 4 °C, 45 min; the final supernatant was concentrated using 100 kDa MWCO centrifugal filters (Millipore, Merck, Darmstadt, Germany) prior to ultracentrifugation (100,000× *g*, 4 °C, 70 min; Optima XE-100, Type 70 Ti rotor). Pelleted nanovesicles were resuspended in PBS (SH30256.01, HyClone, Waltham, WA, USA) under sterile laminar flow conditions. The nanovesicles were resuspended in 10 mL of pre-cooled 1×PBS, followed by a second ultracentrifugation under the same conditions (100,000× *g*, 4 °C, 70 min). After discarding the supernatant, the nanovesicles were resuspended in 100–3000 μL of pre-cooled 1×PBS. The BELNs were stored at −80 °C.

### 2.3. Transmission Electron Microscopy (TEM) and Nanoparticle Tracking Analysis (NTA)

Transmission electron microscopy (TEM): 10 μL of BELNs was aspirated in a Petri dish, and a copper mesh was placed on the droplet and incubated for 1 min. After the liquid was blotted with filter paper, the mesh was stained with 5% phosphotungstic acid for 3 min. The copper mesh was placed under the TEM to observe the morphological structure of BELNs, which was then photographed and recorded.

Nanoparticle tracking analysis (NTA):

The BELNs obtained were used to analyze the size distribution and concentration with a NanoSight LM-10. The samples were diluted 1:100 in PBS (1×) and analyzed, with measurements recorded in triplicate over 60 s video intervals with Detect Threshold 3.

### 2.4. LC-MS-Based Lipidomic Analysis

Sample Pretreatment: Take 200 μL of BELNs, add 400 μL of methyl tert-butyl ether and 80 μL of methanol, and vortex. Centrifuge at 3000× *g* for 15 min. Transfer the methyl tert-butyl ether layer to a new centrifuge tube and dry it with nitrogen. Re-dissolve the sample in 100 μL of dichloromethane–methanol (1:1, *v*/*v*) solution. At the same time, take 5 μL of the sample mixture to prepare the QC sample.

Chromatographic Conditions: Column: Waters HSS T3 (100 × 2.1 mm, 1.8 μm); mobile phases: Phase A—acetonitrile–water (6:4, *v*/*v*) with 10 mM ammonium acetate; Phase B—acetonitrile–isopropanol (1:9, *v*/*v*) with 10 mM ammonium acetate; flow rate: 0.3 mL/min; column temperature: 40 °C; injection volume: 2 μL; elution gradient: 0–4 min, 30% B; 4–22 min, linear increase from 30% to 100% B; 22–22.1 min, rapid decrease from 100% to 30% B; and 22.1–26 min, 30% B. Quality control (QC) samples were interspersed within the sample sequence.

Mass Spectrometry Conditions: Electrospray ionization (ESI) parameters: sheath gas: 60 ARB; auxiliary gas: 10 arb; ion spray voltage: 3000 V (positive)/ −2800 V (negative); source temperature: 350 °C; ion transfer tube temperature: 320 °C; scan mode: full-scan MS^2^; and ionization polarity: positive/negative.

Mass range: 80–1200 *m*/*z*; resolution—70,000 (MS^1^) and 17,500 (MS^2^). Data Pretreatment and Substance Identification: The raw data was processed by LipidSearch software (v4.2) for baseline filtering, retention time correction, peak alignment, peak identification, peak extraction, and integration of lipid molecules. The peak area data of lipid molecules was filtered for 0 values, and lipid molecules with more than 70% 0 values in the expression of all samples were deleted. Then, the data was standardized using the metNor function of the R language MetNormalizer software (v8.0.2) package. The RSD value of the QC sample of lipid molecules was calculated for the standardized data, and lipid molecules with RSD > 30% were filtered out to obtain the pretreated lipid expression.

### 2.5. Protein Determination

#### 2.5.1. Protein Quantification and Analysis

Protein quantification of TELNs was conducted using the BCA assay. Protein separation was performed via standard SDS-PAGE electrophoresis with a 5% stacking gel and 10% separating gel system. Post-electrophoresis, resolved protein bands were subjected to dual staining protocols: Coomassie Brilliant Blue staining followed by silver nitrate staining. Gel imaging was systematically executed under controlled conditions for visualization and archival documentation.

#### 2.5.2. Liquid Chromatography–Tandem Mass Spectrometry (LC-MS/MS) Analysis

BELNs were analyzed by LC—MS/MS using an Easy nLC/Ultimate 3000 high-performance liquid chromatograph (Thermo Scientific) coupled with an Orbitrap Fusion Lumos mass spectrometer (Thermo Scientific). Buffer A consisted of 0.1% formic acid in the water, while Buffer B was 0.08% formic acid in acetonitrile–water (80% acetonitrile). The chromatographic column was equilibrated with 100% Buffer A. The samples were automatically loaded onto the mass spectrometry pre-column and then separated by the analytical column. The mobile phase gradient was as follows: 0–8 min, 5% B; 8–58 min, 10% B; 58–70 min, 24% B; 70–71 min, 95% B; and 71–78 min, 95% B. The total mass spectrometry analysis time was 78 min, and the detection was performed in positive ion mode. The parent ion scan range was set to 300–1400 *m*/*z*. For mass spectrometry parameters, the primary mass spectrometry resolution was 120,000, the AGC target was 5 × 10⁵, the primary Maximum IT was 50 ms, and the dynamic exclusion time was 20.0 s. For the analysis of polypeptides and fragments, the MS2 activation type was HCD, the isolation window was 1.6 *m*/*z*, the number of microscans was 1, the secondary Maximum IT was 35 ms, and the normalized collision energy was 33 eV.

The Gene Ontology (GO) framework was implemented for structured ontological classification of differentially expressed proteins (DEPs) through three hierarchical domains: molecular functionality, biological process involvement, and cellular compartmentalization. Enrichment significance was determined using Fisher’s exact test with Benjamini–Hochberg correction (FDR < 0.05). Concurrently, pathway perturbation analysis was conducted through KEGG Mapper tools (version 97.1), employing hypergeometric distribution models to identify significantly dysregulated metabolic and signaling cascades (*p* < 0.01).

### 2.6. MiRNA Sequencing for BELNs and Data Analysis

Total RNA was isolated using the NGB-58000 Exosome RNA Isolation Kit (Norgen Biotek, Thorold, ON, Canada), and the concentration and purity of the total RNA at 260 nm/280 nm were determined using a UV micro-spectrophotometer (NanoDrop, Thermo Fisher Scientific, USA). The small RNA sequencing library was constructed using the PE150 sequencing protocol, and the quality of the sequencing library was evaluated using fastqc. Sequences were processed using fastp to remove N bases at both ends, perform Q20 filtering, and remove adaptors. The clean sequences were aligned to the Rfam database using the bowtie short-read alignment tool to remove ncRNAs such as rRNA and tRNA and then aligned to the genome. Valid data were obtained by aligning to the mRNA (https://www.ncbi.nlm.nih.gov/genome, accessed on 12 April 2025) database and finally aligned to the miRNA-identified precursors and the genome. To further establish the connection between miRNA and function, GO and KEGG were annotated on the miRNA target genes.

### 2.7. Endothelial Cell Culture and Viability Assessment

Human Umbilical Vein Endothelial Cells (HUVECs) were maintained in Dulbecco’s Modified Eagle Medium (DMEM, Gibco Thermo Fisher Scientific, USA) supplemented with 10% fetal bovine serum (FBS, Gibco, Thermo Fisher Scientific, USA), 100 μg/mL streptomycin, and 100 U/mL penicillin (Solarbio, Beijing, China) under standard culture conditions (37 °C, 5% CO_2_). For experimental modeling, cells were exposed to 250 μM of Palmitic Acid (PA, Sigma, Darmstadt, Germany) dissolved in fatty acid-free BSA (0.5% *w*/*v*). Cellular metabolic activity was quantified using the CCK-8 assay (Bioss, Beijing, China) following manufacturer protocols, with optical density measured at 450 nm (OD 450) using a microplate reader (BioTek Synergy H1, BioTek, Winooski, VT, USA).

### 2.8. Oil Red O Staining and Biochemical Indicator Testing

HUVECs were seeded in 6-well plates and incubated overnight. After treatment, the cells were stained by sequentially adding the oil red O fixative, oil red O staining solution, and Mayer hematoxylin staining solution as per the kit’s protocol (Solarbio, Beijing, China). Finally, the buffer was added, and the cells were imaged under a microscope for examination. In accordance with the instructions of the total cholesterol (TC) and triglyceride (TG) content assay kit (Solarbio, Beijing, China), the effect of BELNs on the TC and TG contents in PA-induced HUVECs was determined using biochemical assays.

### 2.9. Statistical Analysis

Statistical evaluations were conducted using GraphPad Prism 5.0 (v8.0.2, GraphPad Software Inc., San Diego, CA, USA) with one-way ANOVA followed by Tukey’s post hoc test for multi-group comparisons. Significance thresholds were established at * *p* < 0.05 and ** *p* < 0.01.

## 3. Results

### 3.1. The Extraction and Structural Characterization of BELNs

**Figure 1:**
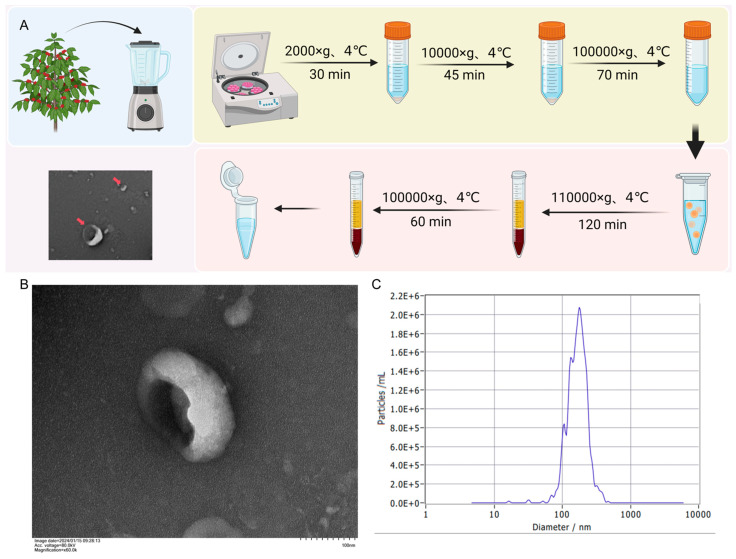
The extraction and structural characterization of BELNs. Created with BioRender.com. (**A**) Flowchart of BELN extraction. (**B**) TEM results showed BELNs were cup-shaped or irregular spherical vesicles. (**C**) The NTA results showed that the detected concentration of BELN particles was 2.1 × 10^13^ particles/mL, and the average diameter was 179.1 nm. The read arrow indicates BELNs.

Ultracentrifugation was used to isolate the BELNs solution from the fruits of *Berberis kaschgarica*, and the BELNs were purified by sucrose density gradient centrifugation (Figure 1A). In this study, its structural characterization was carried out by using transmission electron microscopy and a particle size analyzer. As shown in Figure 1B, BELNs were cup-shaped or irregular spherical vesicles. The NTA results (Figure 1C) showed that the detected concentration of BELNs particles was 2.1 × 10^13^ particles/mL, and the average diameter was 179.1 nm. Similar to the NTA results of previous studies, the diameter was between 100 and 200 nm.

### 3.2. Lipid Composition of BELNs

**Figure 2:**
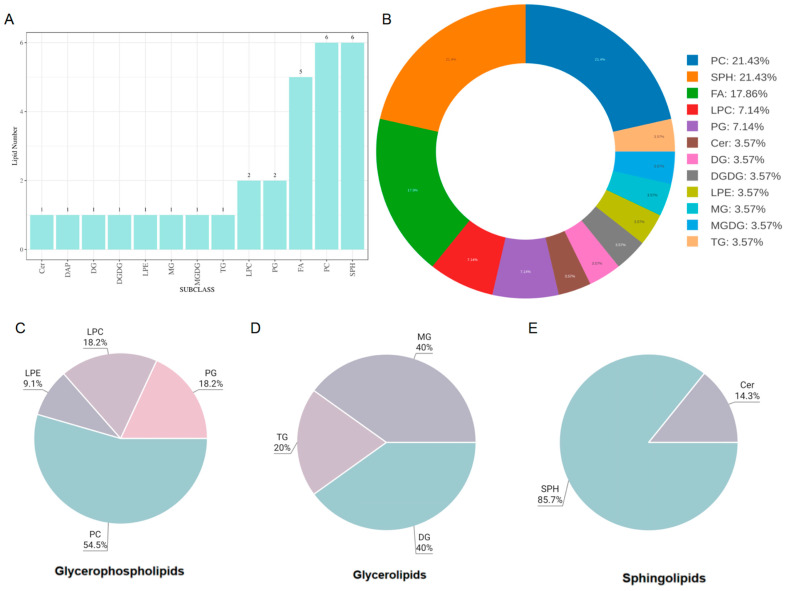
Lipid composition of BELNs. (**A**) The graph shows the 13 lipid classes amount relative to the protein content. (**B**) The graph shows the composition by lipid class, which was defined as the sum of the amount of identified lipid species. (**C**) The graph shows the GP class distribution. The amount of each GP class was expressed as a percentage of the sum of all identified GP species. (**D**) The graph shows the GL class distribution. The amount of each GL class is expressed as a percentage of the sum of all identified GL species. (**E**) The graph shows the SPH class distribution. The amount of each SPH class was expressed as a percentage of the sum of all identified SPH species.

LC-MS analysis of lipids from BELNs provided a dataset of 29 annotated molecular species belonging to 13 lipid classes (Table 1 and Figure 2A).

The lipidomic identification results of BELNs showed that 27 kinds of lipids were identified in BELNs, among which the top five were phosphatidylcholine (PC), sphingomyelin (SPH), fatty acid (FA), phosphatidylglycerol (PG), and lysophosphatidylcholine (LPC), with contents of 21.43%, 21.43%, 17.86%, 7.14%, and 7.14%, respectively (Figure 2 B). We further examined the compositional differences among lipid classes in BELNs (Figure 2 C–E). The results indicated that the composition and proportion of phospholipids in BELNs far exceed those of other lipids, which may reflect the composition and structural characteristics of the phospholipid bilayer-encapsulated vesicles of BELNs.

### 3.3. Proteomic Analyses of BELNs

**Figure 3:**
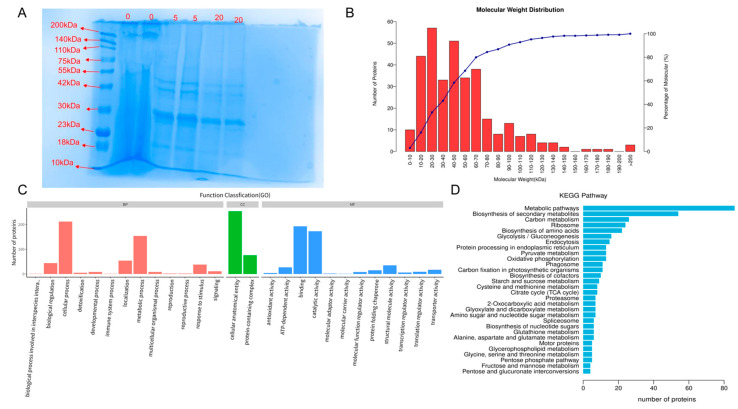
Proteomic analyses of BELNs. (**A**) SDS−PAGE analysis of BELNs. (**B**) Molecular weight distribution. (**C**) GO enrichment analysis. The top GO terms in biological process (BF), cellular component (CC), and molecular function (MF) are displayed. (**D**) KEGG pathway enrichment analysis. The top 30 pathways are presented.

The quantitative BCA assay results showed that each milligram of the BELNs gel precipitate contained 51.35 mg/mL of protein. SDS—PAGE analysis revealed that the proteins in BELNs predominantly had molecular weights below 70 kDa (Figure 3A,B). These findings were in good agreement with previously published results.

The identified proteins are presented in Supplementary Table S1. Gene Ontology (GO) analysis of these proteins showed significant enrichment: 13 proteins were associated with “Biological process,” 2 with “Cellular components,” and 12 with “Molecular function.” For the biological process category, a *p*-value less than 0.05 indicated that multicellular organismal processes, biological regulation, processes involved in interspecies interactions, immune system processes, metabolic processes, and cellular processes were significantly represented. In terms of cellular components, most proteins were linked to cellular anatomical entities and protein-containing complexes. Regarding molecular functions, significant associations were found with protein-folding chaperones, transcription regulator activity, molecular carrier activity, structural molecule activity, transporter activity, and ATP-dependent activity (Figure 3C, Supplementary Table S2).

The KEGG database, which comprehensively documents molecular interaction and reaction pathways, was utilized to determine the relevant biological pathways. By mapping the identified proteins to the KEGG database, 89 enriched pathways were uncovered (Figure 3D, Supplementary Table S3). *p*-value analysis revealed that these proteins were predominantly associated with Metabolic pathways, biosynthesis of secondary metabolites, carbon metabolism, ribosomes, and biosynthesis of amino acids.

### 3.4. Contents of Small RNAs in BELNs

**Figure 4:**
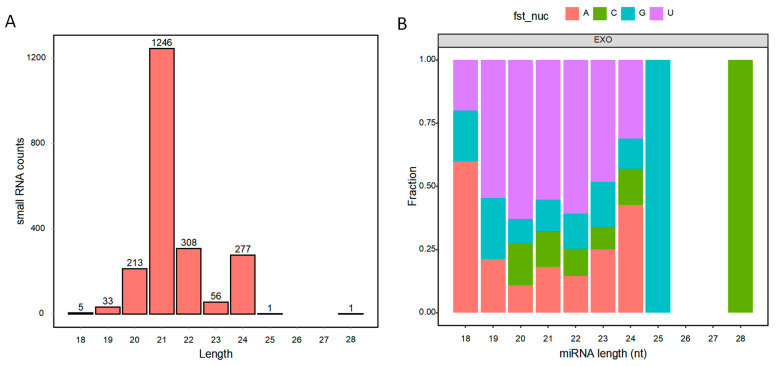
Contents of small RNAs in BELNs. (**A**) sRNA length distribution analysis. (**B**) The first-site preference of miRNA.

The quantitative assay results showed that the RNA yield was 9.5 ng/μg of BELN protein. To characterize the sRNA profiles in BELNs, we first performed size selection and length distribution analysis (Figure 4A). Animal sRNAs typically range from 18 to 35 nt, while plant sRNAs span from 18 to 30 nt, with distinct length preferences reflecting their functional categories. In BELNs, sRNAs predominantly clustered within 20–24 nt, aligning with known miRNA size ranges. We then cross-referenced these sequences against the miRNA-target gene library derived from sequencing data to identify matches. A total of 2140 miRNAs were identified. In-depth analysis revealed the presence of five highly conserved plant microRNAs: miR166, miR156, miR399, miR171, and miR395. The analysis of the first-site preference of miRNA is shown in Figure 4B. Notably, miRNA maturation requires precise enzymatic cleavage, contributing to strong sequence specificity. These findings provided a foundation for further mechanistic studies on miRNA processing and functional validation.

**Figure 5:**
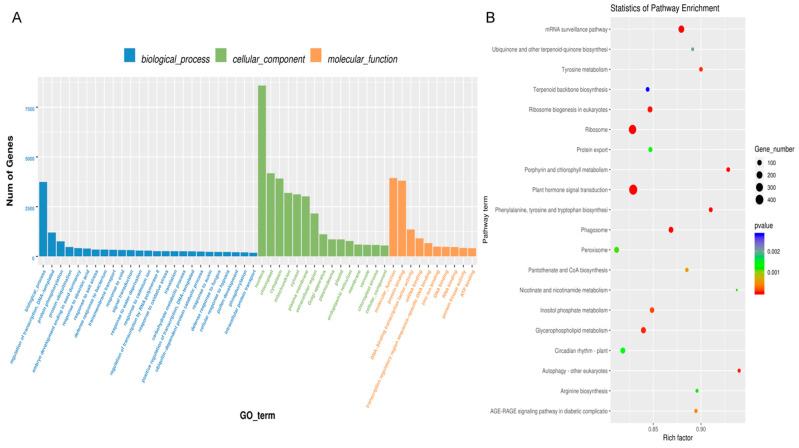
GO and KEGG enrichment analysis. (**A**) GO enrichment analysis. The top GO terms in biological process (BF), cellular component (CC), and molecular function (MF) are displayed. (**B**) KEGG pathway enrichment analysis. The top 20 pathways are presented.

GO and KEGG analyses were conducted to explore the potential biological functions of highly expressed miRNAs in BELNs. The GO analysis (Figure 5A, Supplementary Table S4) categorized the predicted functions of these miRNAs into three main groups. Regarding biological processes, the miRNAs were primarily associated with responses to abscisic acid, cold, and cadmium ions, as well as processes such as embryo development leading to seed dormancy and responses to water deprivation. In the cellular component category, the miRNAs were mainly implicated in the cytosol, plasma membrane, nucleus, cytoplasm, and chloroplast. For molecular functions, they were predominantly involved in activities like protein binding, mRNA binding, transcription regulation, sequence-specific DNA binding, DNA-binding transcription factor activity, and serving as structural components of ribosomes.

Additionally, functional annotation using the KEGG database (Figure 5B, Supplementary Table S5) was carried out. The miRNAs obtained from BELNs participated in various signaling pathways and metabolic processes. The KEGG enrichment scatter plot indicated that these miRNAs were most prominently enriched in the miRNA surveillance, AGE—RAGE, diabetic complications, and MAPK signaling pathways.

### 3.5. BELNs Effectively Enhance Cell Viability and Reduce Lipid Deposition

**Figure 6:**
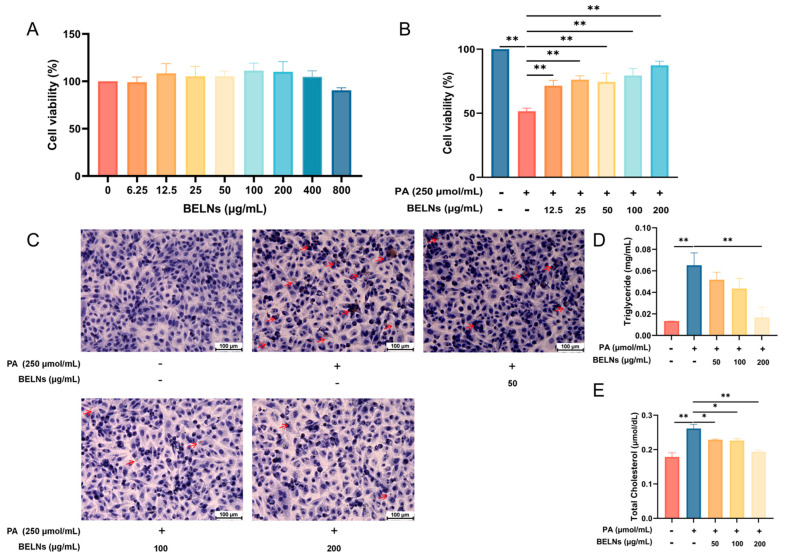
The results of the CCK−8 assay, oil red O staining, and the contents of TG and TC in HUVECs. (**A**,**B**) HUVECs were treated with different concentrations of drugs and then cell viability was assayed by the CCK−8 kit. (**C**) Oil red O staining to analyze lipid deposition in cells treated with different concentrations of drugs. (**D**,**E**) The TC and TG levels were measured and statistically analyzed in PA-induced cells. Error bars represent the average values ± SD (*n* = 3). * represents *p* < 0.05, ** represents *p* < 0.01.

The Cell Counting Kit-8 (CCK8) kit was utilized to explore the effects of BELNs on cell viability and the influence of BELNs on PA-induced lipid impairment in HUVECs. Within the concentration range of 25 to 800 μg/mL, BELNs do not exhibit significant toxicity as evidenced by cell viability assays (Figure 6A). The results presented in Figure 6B of the CCK-8 assay indicated compared with the model group, the cell viability in each BELNs group was improved to varying degrees. Based on the outcomes of the CCK-8 assay, it was affirmed through the oil red O staining assay that the BELNs prominently reduce the intracellular lipid deposition caused by PA (Figure 6C).

Biochemical kits were employed to determine the contents of triglyceride (TG) and total cholesterol (TC) in HUVECs. The findings presented in Figure 6D,E indicated that BELNs led to a substantial decrease in the TG and TC contents in PA-induced cells.

## 4. Discussion

The incorporation of plant-based diets has been widely recognized as a protective dietary strategy for human health. Epidemiological evidence substantiates an inverse correlation between fruit and vegetable consumption and incidence rates of chronic pathologies, including cardiovascular disorders, metabolic syndromes, neoplasms, and age-associated physiological deterioration [16,17,18,19]. Notably, Brondani et al. [20] demonstrated that diets abundant in phytonutrient-rich produce correlate with diminished osteoporotic fracture risks through bone density preservation. While phytochemicals like flavonoid glycosides mediate protective effects [21], isolated supplements show weaker bioefficacy than whole plants, suggesting uncharacterized bioactive complexes and synergies. Emerging research identifies 50–150 nm plant exosome-like nanovesicles (ELNs) as intercellular signaling vesicles, loading regulatory biomolecules (e.g., miR168a, sphingolipids) to modulate innate immunity and cross-kingdom communication [22,23,24]. Mechanistic studies reveal their gastrointestinal tract survivability and cellular internalization capacity. Ju et al. [25] documented ELN traversal through the intestinal mucus barrier and subsequent uptake by murine intestinal stem cells. Particularly compelling are the documented cross-species bioactivities of ELNs derived from Citrus limon (lemon) and Vaccinium spp. (blueberry), which demonstrate therapeutic potential in mammalian systems, thereby positioning fruit-derived nanovesicles as novel biotherapeutic platforms [26,27].

*Berberis kaschgarica*, a wild plant, has fruits abundant in vitamin C and bioactive compounds like anthocyanins and alkaloids. These components endow the fruits with notable biological properties [28]. In our recent studies, we demonstrated that extracts of whole *Berberis kaschgarica* Rupr. or its anthocyanin-rich fraction exhibit antioxidant, hypolipidemic, antiatherosclerotic, and anti-inflammatory effects [29,30,31]. However, due to the narrow natural distribution area and the lack of artificial cultivation techniques, a large-scale collection and processing system has not yet been established. Due to the lack of standardized harvesting, storage, transportation, and deep processing technologies, a large number of mature fruits rot in the natural environment due to not being utilized in time, resulting in a hidden waste of nutritional resources. Developing high-value-added products such as nutritional supplements to improve resource utilization and reduce resource loss caused by technical barriers has become a promising research direction. *Berberis kaschgarica* Rupr. can be categorized as a functional food due to its abundant phytochemicals and vitamins, which offer significant health benefits. In this study, we for the first time report the isolation, purification, and characterization of exosome-like nanovesicles (BELNs) derived from *Berberis kaschgarica* Rupr.

**Figure 7:**
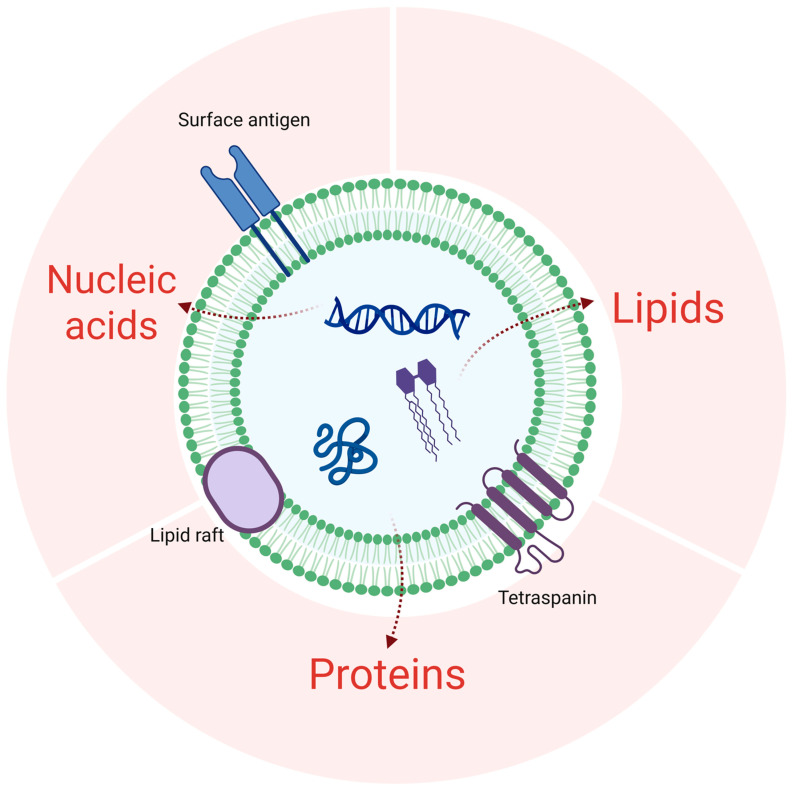
Main components of the BELN structure. Proteins: Surface antigen: involved in the specific recognition between BELNs and target cells, mediates the uptake of BELNs by plant cells, and affects the exertion of their functions; lipids: form the membrane structure of BELNs, maintaining their stability; and nucleic acids: contain the genetic information carried by plant-like nanovesicles, such as mRNA and miRNA. Created with BioRender.com.

BELNs were isolated from *Berberis kaschgarica Rupr.* juice using a traditional differential and ultracentrifugation method, similar to other common PELN isolation techniques. After low-speed centrifugation to remove dead cells, BELNs were obtained by ultracentrifugation and resuspended in PBS. TEM and NTA analysis confirmed that BELNs were membrane-enclosed nanovesicles, with an average size of 179.1 nm, which is comparable to those from other edible plants and fruits [32,33,34,35,36]. To understand how BELNs affect mammalian cells, it is crucial to know their biochemical composition. Therefore, we conducted comprehensive lipidomic, metabolomic, and proteomic analyses (Figure 7). The findings from these characterizations offer new insights into the potential of BELNs as nutraceuticals and therapeutic agents.

Lipids are essential for maintaining ELNs’ bilayer membrane integrity, stability, cellular uptake, and biological activities [25]. In BELNs, 30 lipids were identified, with phosphatidylcholine (PC, 21.43%), sphingomyelin (SPH, 21.43%), fatty acid (FA, 17.86%), phosphatidylglycerol (PG, 7.14%), and lysophosphatidylcholine (LPC, 7.14%) as major components. PC ensures membrane stability, facilitates fusion with target cells, and enhances drug delivery [37]. SPH in lipid rafts enables cholesterol-mediated targeting and modulates immune responses via sphingolipid metabolites [38,39]. FAs (e.g., linoleic acid) serve as energy substrates, enhance membrane flexibility, and improve tissue penetration [40]. PG’s anionic charge promotes electrostatic adhesion to pathological sites and synergizes with antimicrobials [41]. LPC enhances intracellular drug release and recruits immune cells for targeted immunomodulation [42]. PELNs’ surface lipids (PC) ensure biocompatibility, while lipid raft organization (SPH) and charge characteristics (PG) optimize tissue-specific targeting. Their hydrophobic domains enable lipophilic drug encapsulation, and high PC/SPH content ensures stability. Despite their potential as next-generation nanocarriers, they require engineering and safety validation to address translational challenges.

Proteins, as crucial biomolecules that convey biological information and are indispensable for the functionality of ELNs, can be classified into transmembrane and other plasma membrane-associated varieties in plant-derived ELNs. Our research outcomes indicated that BELNs primarily encompassed cytoplasmic proteins, while the identification of membrane transport proteins such as water and chloride channel proteins was relatively limited. By conducting a *p*-value analysis, it was determined that the proteins in BELNs are mainly implicated in metabolic pathways, biosynthesis of secondary metabolites, carbon metabolism, ribosome-related processes, and amino acid biosynthesis. Nevertheless, these proteins do not possess the requisite universality to act as a marker for PELNs. Therefore, extensive and in-depth research is currently requisite to comprehensively identify the proteins in diverse plant-derived ELNs and to elucidate their roles in biological and pharmacological activities.

ELN cargos originate from specific tissues and cells, carrying unique combinations of miRNAs, proteins, and secondary metabolites [43]. In the analyzed ELNs, several miRNAs were detected, including members of the miR166, miR156, miR399, miR171, and miR395 families. These miRNAs integrate endogenous signals with environmental cues—both biotic and abiotic—to regulate various plant growth and physiological processes. Additionally, their cross-kingdom regulatory potential was explored. MiR166 and its mode of action have been reported in multiple plant species [44]. For instance, miR166 from Moringa oleifera Lam seeds was predicted to target genes such as BCL2, IL2RA, TNF, and VAV1, which are involved in the cell cycle, apoptosis, immune response, and HIV pathogenesis, respectively [45]. MiR156 is a highly conserved miRNA across the plant kingdom. Li et al. demonstrated that plant MIR156 regulates intestinal cell proliferation in vitro and in vivo. In mice, a diet rich in plant miRNAs or synthetic MIR156 increased MIR156 levels and inhibited the Wnt/β-catenin signaling pathway. Bioinformatics and luciferase assays identified Wnt10b as a target of MIR156. In porcine jejunum epithelial (IPEC—J2) cells, MIR156 suppressed cell proliferation by downregulating Wnt10b protein levels and upregulating β-catenin phosphorylation, an effect reversed by lithium chloride or an MIR156 inhibitor [46]. Moreover, ginseng-derived MiR156 was shown to enhance macrophage proliferation, phagocytosis, and immunomodulatory functions [47]. Previous work has shown that emerging evidence suggests that miR171vr, a sequence variant of plant-derived miR171, demonstrates putative bioactivity in human embryonic kidney 293 (HEK293) cell models. Preliminary evidence implies that miR171vr, whether introduced through diet or as a supplement in gene therapies, might potentially have an impact on human gene expression, particularly in the treatment of disorders where GNA12 is overexpressed (such as oral cancer, breast, and prostate adenocarcinoma) or mTOR kinase is downregulated (for example, obesity, type 2 diabetes, and neurodegeneration) [48]. Thus, these miRNAs, by dietary intake, could target human genes and influence a range of immune- and metabolic-related biological pathways.

Endothelial functional impairment is an early event of AS. When endothelial cells are exposed to risk factors such as free fatty acids, cholesterol crystals, inflammatory stimuli, and blood flow changes, they are activated, leading to an increase in endothelial cell permeability and the release of inflammatory factors. Monocytes are recruited to attach to the activated endothelial cells, and at the same time, more lipids can pass through the endothelial cells into the inner wall of the artery, resulting in lipid accumulation [49]. In this study, we found that under the stimulation of PA, the viability of HUVECs decreased, and after treatment with BELNs, the cell viability was significantly enhanced. During the atherosclerotic lesion process, the elevated levels of TC and TG synthesized by the liver are the main risk factors for atherosclerosis [50]. TC is prone to deposit on the arterial wall and form plaques with the proliferation of arterial wall cells. After stimulation with PA, the intracellular lipid deposition, TG, and TC contents in HUVECs increased. However, after treatment with BELNs, the intracellular lipid deposition and the contents of TG and TC significantly decreased, demonstrating that BELNs could reduce intracellular lipid deposition.

These findings suggest BELNs’ potential to modulate fundamental biological processes, particularly in immune regulation and bioactive molecule delivery. To validate their utility as nutraceutical delivery vehicles, critical assessments of their stability in physiological fluids and resilience under simulated gastric conditions are essential. Ongoing research focuses on characterizing their functional properties to substantiate applications in dietary supplements and functional foods.

This study has several notable limitations. First, although this research identified multiple components in PELNs, a deep-seated understanding of their specific functions and underlying mechanisms remains lacking. The various components within BELNs may exhibit complex interaction patterns. However, this study did not conduct an in-depth analysis of these interactions, making it impossible to comprehensively grasp the overall functions of ELNs. Second, the proposed effects of these cross-kingdom miRNAs are currently theoretical and require further experimental verification. At present, there is a lack of direct evidence from in vitro or in vivo experiments to validate whether these miRNAs can effectively regulate target genes in recipient organisms and exert expected biological effects. Third, this study did not incorporate positive control drugs with confirmed efficacy in the field or other types of exosomal vesicles as comparative references. Such efforts will contribute to a more thorough understanding of the roles and application potentials of plant ELNs.

## 5. Conclusions

In this study, BELNs were isolated, purified, and characterized from *Berberis kaschgarica* Rupr. juice. A systematic and comprehensive physicochemical analysis of the BELNs showed that they were rich in lipids and proteins. Moreover, these Berberis kaschgarica Rupr.-derived BELNs contain small RNAs. Their potential effects on human cells could lead to novel nutraceutical strategies and stimulate discussions about the cross-kingdom activity of miRNAs. The nature of the biomolecules identified in BELNs suggests that they could be used in the food and nutraceutical industries. This indicates a potential medical application for ELNs in regulating fundamental biological processes in the human body, including the regulation of lipids and delivering bioactive molecules. This study represents the inaugural combination of multi-omics to analyze the characteristics and nature of the BELNs, which furnishes a significant scientific foundation for the subsequent advancement and exploitation of *Berberis kaschgarica* Rupr.

## Figures and Tables

**Table 1:** The detected lipids by LC-MS from BELNs.

Metabolite	Retention Time (min)	m/z	Adducts
DG(16:0_16:0)	17.99592699	586.5405005	M + NH4
MGDG(18:3_18:3)	13.91099355	797.5174215	M + Na
LPE(18:2)	2.651805773	476.2782655	M−H
LPC(16:0)	3.221360685	540.3306955	M + HCOO
DAP(O-8:0_O-8:0)	11.19310263	391.2842865	M + H
TG(O-15:0_18:0_3:0)	19.37484361	642.6031005	M + NH4
SPH(d14:1)	2.143380594	244.2271055	M + H
LPC(18:1)	3.561631005	522.3554185	M + H
Cer(d16:0_16:0)	17.24053772	512.5037205	M + H
PC(34:3)	14.67353089	756.5537835	M + H
PC(34:2)	15.43213762	758.5694335	M + H
PC(34:1)	16.21210249	760.5850835	M + H
PC(36:4)	14.79537964	782.5694335	M + H
SPH(d15:0)	1.594016317	244.2634905	M + H
PC(36:3)	15.55398637	784.5850835	M + H
PC(36:2)	16.3055966	786.6007335	M + H
SPH(d14:0)	1.127579401	246.2427555	M + H
SPH(d17:0)	1.898047996	288.2897055	M + H
SPH(d18:0)	2.33114669	302.3053555	M + H
SPH(d20:0)	3.980294343	330.3366555	M + H
MG(O-15:2)	2.444098446	299.2580715	M + H
FA(16:0)	8.101046132	255.2329535	M−H
FA(22:0)	16.2752598	339.3268535	M−H
FA(26:0)	18.18207962	395.3894535	M−H
FA(28:0)	18.95159637	423.4207535	M−H
FA(30:0)	19.44821288	451.4520535	M−H
PG(25:2_22:7)	12.55670308	913.5964115	M−H
PG(25:1_22:7)	14.89752817	915.6120615	M−H
DGDG(20:0_14:2)	12.73955966	915.6050485	M−H

## Data Availability

The original contributions presented in this study are included in the article. Further inquiries can be directed to the corresponding author(s).

## Supplementary Materials

The following supporting information can be downloaded at https://www.mdpi.com/article/10.3390/biology14060726/s1: Table S1. Identification details of the proteins identified in BELNs; Supplementary Table S2. Details of biological processes, cellular components, and molecular functions of proteins identified from BELNs; Supplementary Table S3. KEGG pathway analysis of proteins identified from BELNs; Supplementary Table S4. Details of biological processes, cellular components, and molecular functions of miRNAs identified from BELNs; Supplementary Table S5. KEGG pathway analysis of miRNAs identified from BELNs.

## Abbreviations

The following abbreviations are used in this manuscript:

BELNs	*Berberis kaschgarica* Rupr.-derived exosome-like nanovesicles
PC	Phosphatidylcholine
SPH	Sphingomyelin
FA	Fatty acid
PG	Phosphatidylglycerol
LPC	Lysophosphatidylcholine
ELNs	Exosome-like nanoplants
TEM	Transmission electron microscopy
NTA	Nanoparticle tracking analysis
LC-MS/MS	Liquid Chromatography–Tandem Mass Spectrometry
GO	Gene Ontology
KEGG	Kyoto Encyclopedia of Genes and Genomes
