# Supplementary material for: Isolation and Bioactive Characterization of Berberis kaschgarica Rupr-Derived Exosome-Like Nanovesicles: Exploring Therapeutic Potential in Atherosclerosis Pathogenesis
Source: Biology (Basel). 2025 Jun 19;14(6):726. doi: 10.3390/biology14060726 (PMC12189238; doi:10.3390/biology14060726)
Supplement: Supplementary file 1 [file biology-14-00726-s001.zip › Supplementary Tables S2-S5.pdf]

Supplementary Table S2 Details of Biological processes, cellular components and molecular functions of proteins identified from BELNs

GO_ID	class	term	number
GO:0032501	biological_process	multicellular organismal process	8
GO:0023052	biological_process	signaling	11
GO:0044183	molecular_function	protein folding chaperone	15
GO:0050896	biological_process	response to stimulus	38
GO:0005488	molecular_function	binding	193
GO:0022414	biological_process	reproductive process	2
GO:0140110	molecular_function	transcription regulator activity	6
GO:0065007	biological_process	biological regulation	44
GO:0110165	cellular_component	cellular anatomical entity	254
GO:0140104	molecular_function	molecular carrier activity	1
GO:0032502	biological_process	developmental process	8
GO:0005198	molecular_function	structural molecule activity	35
GO:0005215	molecular_function	transporter activity	17
GO:0032991	cellular_component	protein-containing complex	76
GO:0051179	biological_process	localization	54
GO:0140657	molecular_function	ATP-dependent activity	27
GO:0044419	biological_process	biological process involved in interspecies interaction between organisms	1
GO:0045182	molecular_function	translation regulator activity	9
GO:0002376	biological_process	immune system process	1
GO:0098754	biological_process	detoxification	5
GO:0008152	biological_process	metabolic process	154
GO:0098772	molecular_function	molecular function regulator activity	8
GO:0003824	molecular_function	catalytic activity	173
GO:0009987	biological_process	cellular process	212
GO:0000003	biological_process	reproduction	2
GO:0060090	molecular_function	molecular adaptor activity	2
GO:0016209	molecular_function	antioxidant activity	4

Supplementary Table S3 KEGG Pathway analysis of proteins identified from BELNs

ko_id	ko_description	pathway_protein_number
ko00010	Glycolysis / Gluconeogenesis	16
ko00020	Citrate cycle (TCA cycle)	8
ko00030	Pentose phosphate pathway	5
ko00040	Pentose and glucuronate interconversions	4
ko00051	Fructose and mannose metabolism	4
ko00052	Galactose metabolism	3
ko00053	Ascorbate and aldarate metabolism	4
ko00061	Fatty acid biosynthesis	1
ko00071	Fatty acid degradation	1
ko00190	Oxidative phosphorylation	13

ko00195	Photosynthesis	3
ko00220	Arginine biosynthesis	2
ko00230	Purine metabolism	4
ko00240	Pyrimidine metabolism	2
ko00250	Alanine, aspartate and glutamate metabolism	6
ko00260	Glycine, serine and threonine metabolism	5
ko00270	Cysteine and methionine metabolism	8
ko00280	Valine, leucine and isoleucine degradation	2
ko00290	Valine, leucine and isoleucine biosynthesis	1
ko00310	Lysine degradation	2
ko00330	Arginine and proline metabolism	3
ko00350	Tyrosine metabolism	3
ko00360	Phenylalanine metabolism	1
ko00380	Tryptophan metabolism	2
ko00400	Phenylalanine, tyrosine and tryptophan biosynthesis	1
ko00410	beta-Alanine metabolism	3
ko00430	Taurine and hypotaurine metabolism	2
ko00450	Selenocompound metabolism	2
ko00460	Cyanoamino acid metabolism	3
ko00480	Glutathione metabolism	6
ko00500	Starch and sucrose metabolism	9
ko00520	Amino sugar and nucleotide sugar metabolism	7
ko00561	Glycerolipid metabolism	2
ko00562	Inositol phosphate metabolism	2
ko00564	Glycerophospholipid metabolism	5
ko00565	Ether lipid metabolism	1
ko00592	alpha-Linolenic acid metabolism	2
ko00620	Pyruvate metabolism	13
ko00630	Glyoxylate and dicarboxylate metabolism	7
ko00640	Propanoate metabolism	3
ko00650	Butanoate metabolism	2
ko00670	One carbon pool by folate	1
ko00710	Carbon fixation in photosynthetic organisms	11
ko00730	Thiamine metabolism	2
ko00750	Vitamin B6 metabolism	1
ko00770	Pantothenate and CoA biosynthesis	2
ko00785	Lipoic acid metabolism	3
ko00910	Nitrogen metabolism	1
ko00920	Sulfur metabolism	1
ko00940	Phenylpropanoid biosynthesis	1
ko00950	Isoquinoline alkaloid biosynthesis	1
ko00960	Tropane, piperidine and pyridine alkaloid biosynthesis	1

ko00970	Aminoacyl-tRNA biosynthesis	4
ko00999	Biosynthesis of various plant secondary metabolites	2
ko01100	Metabolic pathways	86
ko01110	Biosynthesis of secondary metabolites	54
ko01200	Carbon metabolism	26
ko01210	2-Oxocarboxylic acid metabolism	7
ko01212	Fatty acid metabolism	1
ko01230	Biosynthesis of amino acids	22
ko01232	Nucleotide metabolism	3
ko01240	Biosynthesis of cofactors	10
ko01250	Biosynthesis of nucleotide sugars	6
ko03008	Ribosome biogenesis in eukaryotes	1
ko03010	Ribosome	24
ko03013	Nucleocytoplasmic transport	4
ko03015	mRNA surveillance pathway	4
ko03018	RNA degradation	4
ko03040	Spliceosome	6
ko03050	Proteasome	7
ko03060	Protein export	2
ko03082	ATP-dependent chromatin remodeling	1
ko03083	Polycomb repressive complex	2
ko03250	Viral life cycle - HIV-1	2
ko04016	MAPK signaling pathway - plant	3
ko04070	Phosphatidylinositol signaling system	3
ko04075	Plant hormone signal transduction	2
ko04120	Ubiquitin mediated proteolysis	1
ko04122	Sulfur relay system	1
ko04130	SNARE interactions in vesicular transport	2
ko04141	Protein processing in endoplasmic reticulum	13
ko04144	Endocytosis	15
ko04145	Phagosome	11
ko04146	Peroxisome	3
ko04148	Efferocytosis	2
ko04626	Plant-pathogen interaction	4
ko04814	Motor proteins	5

Supplementary Table S4 Details of Biological processes, cellular components and molecular functions of miRNA identified from BELNs

GO Id	Function	GO_term	Pvalue
GO:0005515	molecular_function	protein binding	1.68E-199
GO:0005829	cellular_component	cytosol	1.25E-169
GO:0005886	cellular_component	plasma membrane	2.19E-126
GO:0005634	cellular_component	nucleus	1.97E-122
GO:0005737	cellular_component	cytoplasm	3.05E-114

GO:0009507	cellular_component	chloroplast	2.57E-108
GO:0003729	molecular_function	mRNA binding	5.54E-62
GO:0005794	cellular_component	Golgi apparatus	3.38E-61
GO:0009536	cellular_component	plastid	1.86E-57
GO:0009941	cellular_component	chloroplast envelope	1.00E-40
GO:0009570	cellular_component	chloroplast stroma	2.25E-37
GO:0005783	cellular_component	endoplasmic reticulum	3.57E-34
GO:0009506	cellular_component	plasmodesma	6.37E-34
GO:0000976	molecular_function	transcription regulatory region sequence-specific DNA binding	8.47E-30
GO:0005773	cellular_component	vacuole	4.34E-26
GO:0005774	cellular_component	vacuolar membrane	1.84E-25
GO:0009737	biological_process	response to abscisic acid	1.94E-24
GO:0003700	molecular_function	DNA-binding transcription factor activity	8.95E-23
GO:0005730	cellular_component	nucleolus	1.94E-20
GO:0005768	cellular_component	endosome	5.46E-20
GO:0009409	biological_process	response to cold	5.92E-20
GO:0003735	molecular_function	structural constituent of ribosome	3.83E-19
GO:0046686	biological_process	response to cadmium ion	8.77E-18
GO:0009793	biological_process	embryo development ending in seed dormancy	6.61E-17
GO:0005802	cellular_component	trans-Golgi network	8.62E-17
GO:0003677	molecular_function	DNA binding	1.04E-16
GO:0004674	molecular_function	protein serine/threonine kinase activity	2.65E-16
GO:0022626	cellular_component	cytosolic ribosome	7.19E-16
GO:0009535	cellular_component	chloroplast thylakoid membrane	8.26E-16
GO:0009414	biological_process	response to water deprivation	2.31E-15
GO:0006886	biological_process	intracellular protein transport	6.82E-15
GO:0009579	cellular_component	thylakoid	7.78E-15
GO:0071456	biological_process	cellular response to hypoxia	6.52E-14
GO:0004672	molecular_function	protein kinase activity	9.14E-14
GO:0042742	biological_process	defense response to bacterium	1.04E-13
GO:0009534	cellular_component	chloroplast thylakoid	1.60E-13
GO:0006355	biological_process	regulation of transcription, DNA-templated	2.07E-13
GO:0009651	biological_process	response to salt stress	4.90E-13
GO:0016020	cellular_component	membrane	1.79E-12
GO:0006970	biological_process	response to osmotic stress	3.74E-12
GO:0006468	biological_process	protein phosphorylation	4.35E-12
GO:0042803	molecular_function	protein homodimerization activity	9.26E-12
GO:0005524	molecular_function	ATP binding	1.21E-11
GO:0048046	cellular_component	apoplast	2.09E-11
GO:0005507	molecular_function	copper ion binding	1.01E-10
GO:0003723	molecular_function	RNA binding	1.32E-10

GO:0009733	biological_process	response to auxin	1.65E-10
GO:0016192	biological_process	vesicle-mediated transport	1.78E-10
GO:0045893	biological_process	positive regulation of transcription, DNA-templated	5.15E-10
GO:0042802	molecular_function	identical protein binding	5.92E-10
GO:0005516	molecular_function	calmodulin binding	1.68E-09
GO:0006979	biological_process	response to oxidative stress	5.57E-09
GO:0016757	molecular_function	transferase activity, transferring glycosyl groups	6.83E-09
GO:0009658	biological_process	chloroplast organization	7.12E-09
GO:0006457	biological_process	protein folding	7.96E-09
GO:0048364	biological_process	root development	8.85E-09
GO:0005777	cellular_component	peroxisome	1.09E-08
GO:0009555	biological_process	pollen development	1.76E-08
GO:0016887	molecular_function	ATPase activity	2.13E-08
GO:0005509	molecular_function	calcium ion binding	4.54E-08
GO:0005789	cellular_component	endoplasmic reticulum membrane	4.62E-08
GO:0007623	biological_process	circadian rhythm	5.89E-08
GO:0043565	molecular_function	sequence-specific DNA binding	5.96E-08
GO:0008017	molecular_function	microtubule binding	6.56E-08
GO:0005618	cellular_component	cell wall	1.09E-07
GO:0046777	biological_process	protein autophosphorylation	1.20E-07
GO:0010287	cellular_component	plastoglobule	1.89E-07
GO:0003924	molecular_function	GTPase activity	2.01E-07
GO:0009631	biological_process	cold acclimation	2.57E-07
GO:0006281	biological_process	DNA repair	2.70E-07
GO:0005797	cellular_component	Golgi medial cisterna	3.42E-07
GO:0018105	biological_process	peptidyl-serine phosphorylation	5.26E-07
GO:0006633	biological_process	fatty acid biosynthetic process	5.62E-07
GO:0009739	biological_process	response to gibberellin	5.63E-07
GO:0022627	cellular_component	cytosolic small ribosomal subunit	6.16E-07
GO:0009826	biological_process	unidimensional cell growth	6.74E-07
GO:0080167	biological_process	response to karrikin	6.74E-07
GO:0022625	cellular_component	cytosolic large ribosomal subunit	8.27E-07
GO:0099503	cellular_component	secretory vesicle	8.32E-07
GO:0003697	molecular_function	single-stranded DNA binding	9.48E-07
GO:0010114	biological_process	response to red light	1.41E-06
GO:0016491	molecular_function	oxidoreductase activity	1.74E-06
GO:0000138	cellular_component	Golgi trans cisterna	1.78E-06
GO:0010119	biological_process	regulation of stomatal movement	2.17E-06
GO:0045892	biological_process	negative regulation of transcription, DNA-templated	2.61E-06
GO:0009505	cellular_component	plant-type cell wall	3.01E-06
GO:0050832	biological_process	defense response to fungus	3.15E-06

GO:0009416	biological_process	response to light stimulus	3.26E-06
GO:0000502	cellular_component	proteasome complex	3.29E-06
GO:0015979	biological_process	photosynthesis	3.29E-06
GO:0005874	cellular_component	microtubule	3.43E-06
GO:0000977	molecular_function	RNA polymerase II regulatory region sequence-specific DNA binding	3.44E-06
GO:0051082	molecular_function	unfolded protein binding	4.39E-06
GO:0009706	cellular_component	chloroplast inner membrane	4.85E-06
GO:0043621	molecular_function	protein self-association	5.54E-06
GO:0006334	biological_process	nucleosome assembly	6.26E-06
GO:0043022	molecular_function	ribosome binding	6.26E-06
GO:0008270	molecular_function	zinc ion binding	7.00E-06
GO:0005747	cellular_component	mitochondrial respiratory chain complex I	8.27E-06
GO:0001666	biological_process	response to hypoxia	8.45E-06
GO:0010228	biological_process	vegetative to reproductive phase transition of meristem	1.02E-05
GO:0042538	biological_process	hyperosmotic salinity response	1.08E-05
GO:0005635	cellular_component	nuclear envelope	1.21E-05
GO:0007018	biological_process	microtubule-based movement	1.24E-05
GO:0016787	molecular_function	hydrolase activity	1.27E-05
GO:0010089	biological_process	xylem development	1.54E-05
GO:0048316	biological_process	seed development	1.94E-05
GO:0009408	biological_process	response to heat	1.94E-05
GO:0080008	cellular_component	Cul4-RING E3 ubiquitin ligase complex	2.05E-05
GO:0005743	cellular_component	mitochondrial inner membrane	2.39E-05
GO:0009705	cellular_component	plant-type vacuole membrane	2.79E-05
GO:0009611	biological_process	response to wounding	2.89E-05
GO:0030170	molecular_function	pyridoxal phosphate binding	2.96E-05
GO:0030176	cellular_component	integral component of endoplasmic reticulum membrane	3.09E-05
GO:0031977	cellular_component	thylakoid lumen	4.04E-05
GO:0045087	biological_process	innate immune response	4.27E-05
GO:0000398	biological_process	mRNA splicing, via spliceosome	4.88E-05
GO:0106307			4.89E-05
GO:0106306			4.89E-05
GO:0006631	biological_process	fatty acid metabolic process	5.39E-05
GO:0009965	biological_process	leaf morphogenesis	5.42E-05
GO:0009723	biological_process	response to ethylene	6.15E-05
GO:0006351	biological_process	transcription, DNA-templated	6.50E-05
GO:0035556	biological_process	intracellular signal transduction	6.79E-05
GO:0005198	molecular_function	structural molecule activity	6.89E-05
GO:0009504	cellular_component	cell plate	7.11E-05
GO:0000137	cellular_component	Golgi cis cisterna	7.11E-05
GO:0009911	biological_process	positive regulation of flower development	7.11E-05

GO:0010200	biological_process	response to chitin	7.34E-05
GO:0009860	biological_process	pollen tube growth	8.73E-05
GO:0051607	biological_process	defense response to virus	8.86E-05
GO:0003824	molecular_function	catalytic activity	9.23E-05
GO:0010090	biological_process	trichome morphogenesis	9.38E-05
GO:0006952	biological_process	defense response	9.54E-05
GO:0006364	biological_process	rRNA processing	9.67E-05
GO:0003690	molecular_function	double-stranded DNA binding	1.05E-04
GO:0007015	biological_process	actin filament organization	1.15E-04
GO:0009958	biological_process	positive gravitropism	1.24E-04
GO:0008219	biological_process	cell death	1.25E-04
GO:0010091	biological_process	trichome branching	1.25E-04
GO:0042651	cellular_component	thylakoid membrane	1.25E-04
GO:0005938	cellular_component	cell cortex	1.25E-04
GO:0048366	biological_process	leaf development	1.29E-04
GO:0051015	molecular_function	actin filament binding	1.29E-04
GO:0006470	biological_process	protein dephosphorylation	1.31E-04
GO:0005525	molecular_function	GTP binding	1.31E-04
GO:0009626	biological_process	plant-type hypersensitive response	1.49E-04
GO:0010224	biological_process	response to UV-B	1.54E-04
GO:0009751	biological_process	response to salicylic acid	1.62E-04
GO:0009753	biological_process	response to jasmonic acid	1.62E-04
GO:0048868	biological_process	pollen tube development	1.63E-04
GO:0046982	molecular_function	protein heterodimerization activity	1.65E-04
GO:0004693	molecular_function	cyclin-dependent protein serine/threonine kinase activity	1.69E-04
GO:0006342	biological_process	chromatin silencing	1.69E-04
GO:0009553	biological_process	embryo sac development	2.00E-04
GO:0006891	biological_process	intra-Golgi vesicle-mediated transport	2.14E-04
GO:0009853	biological_process	photorespiration	2.14E-04
GO:0003680	molecular_function	AT DNA binding	2.28E-04
GO:0031966	cellular_component	mitochondrial membrane	2.28E-04
GO:0009789	biological_process	positive regulation of abscisic acid-activated signaling pathway	2.46E-04
GO:0010027	biological_process	thylakoid membrane organization	2.46E-04
GO:1990939	molecular_function	ATP-dependent microtubule motor activity	2.46E-04
GO:0010286	biological_process	heat acclimation	2.46E-04
GO:0043622	biological_process	cortical microtubule organization	2.49E-04
GO:0002181	biological_process	cytoplasmic translation	2.49E-04
GO:0005484	molecular_function	SNAP receptor activity	2.49E-04
GO:0042752	biological_process	regulation of circadian rhythm	2.82E-04
GO:0005770	cellular_component	late endosome	2.82E-04
GO:0032502	biological_process	developmental process	2.82E-04

GO:0030154	biological_process	cell differentiation	2.92E-04
GO:0071944	cellular_component	cell periphery	3.08E-04
GO:0005856	cellular_component	cytoskeleton	3.08E-04
GO:0000027	biological_process	ribosomal large subunit assembly	3.08E-04
GO:0009630	biological_process	gravitropism	3.14E-04
GO:0010218	biological_process	response to far red light	3.22E-04
GO:0009735	biological_process	response to cytokinin	3.56E-04
GO:0004175	molecular_function	endopeptidase activity	3.71E-04
GO:0045489	biological_process	pectin biosynthetic process	3.71E-04
GO:0030163	biological_process	protein catabolic process	3.71E-04
GO:0000413	biological_process	protein peptidyl-prolyl isomerization	3.71E-04
GO:0010029	biological_process	regulation of seed germination	4.00E-04
GO:0048481	biological_process	plant ovule development	4.00E-04
GO:0009910	biological_process	negative regulation of flower development	4.00E-04
GO:2000022	biological_process	regulation of jasmonic acid mediated signaling pathway	4.16E-04
GO:0006397	biological_process	mRNA processing	4.16E-04
GO:0009638	biological_process	phototropism	4.16E-04
GO:0016021	cellular_component	integral component of membrane	4.25E-04
GO:0009640	biological_process	photomorphogenesis	4.69E-04
GO:0006888	biological_process	endoplasmic reticulum to Golgi vesicle-mediated transport	5.31E-04
GO:0012505	cellular_component	endomembrane system	5.38E-04
GO:0000287	molecular_function	magnesium ion binding	5.62E-04
GO:0048767	biological_process	root hair elongation	5.63E-04
GO:0000932	cellular_component	P-body	5.85E-04
GO:0009791	biological_process	post-embryonic development	6.40E-04
GO:0006635	biological_process	fatty acid beta-oxidation	6.40E-04
GO:0051539	molecular_function	4 iron, 4 sulfur cluster binding	6.47E-04
GO:0009627	biological_process	systemic acquired resistance	7.07E-04
GO:0031347	biological_process	regulation of defense response	7.07E-04
GO:0051301	biological_process	cell division	7.14E-04
GO:0031410	cellular_component	cytoplasmic vesicle	7.57E-04
GO:0008353	molecular_function	RNA polymerase II CTD heptapeptide repeat kinase activity	7.57E-04
GO:0005839	cellular_component	proteasome core complex	7.57E-04
GO:0010075	biological_process	regulation of meristem growth	7.57E-04
GO:0016607	cellular_component	nuclear speck	8.85E-04
GO:0004712	molecular_function	protein serine/threonine/tyrosine kinase activity	8.87E-04
GO:0000139	cellular_component	Golgi membrane	8.87E-04
GO:0048510	biological_process	regulation of timing of transition from vegetative to reproductive phase	8.89E-04

Supplementary Table S5 KEGG Pathway analysis of miRNA identified from BELNs

Pathway Id	pathway description	Pvalue
ko04075	Plant hormone signal transduction	1.23E-12
ko03015	mRNA surveillance pathway	1.36E-10
ko03010	Ribosome	2.83E-10
ko04145	Phagosome	2.91E-06
ko00860	Porphyrin and chlorophyll metabolism	3.02E-06
ko00400	Phenylalanine, tyrosine and tryptophan biosynthesis	7.39E-06
ko03008	Ribosome biogenesis in eukaryotes	2.38E-05
ko04136	Autophagy - other eukaryotes	3.25E-05
ko00564	Glycerophospholipid metabolism	4.15E-05
ko00350	Tyrosine metabolism	6.07E-05
ko00562	Inositol phosphate metabolism	1.33E-04
ko04933	AGE-RAGE signaling pathway in diabetic complications	4.52E-04
ko00770	Pantothenate and CoA biosynthesis	5.79E-04
ko04146	Peroxisome	1.13E-03
ko00760	Nicotinate and nicotinamide metabolism	1.19E-03
ko00220	Arginine biosynthesis	1.23E-03
ko03060	Protein export	1.38E-03
ko04712	Circadian rhythm - plant	1.41E-03
ko00130	Ubiquinone and other terpenoid-quinone biosynthesis	2.03E-03
ko00900	Terpenoid backbone biosynthesis	2.81E-03
ko00730	Thiamine metabolism	3.01E-03
ko00061	Fatty acid biosynthesis	3.02E-03
ko04070	Phosphatidylinositol signaling system	3.27E-03
ko04130	SNARE interactions in vesicular transport	3.79E-03
ko00071	Fatty acid degradation	3.86E-03
ko00510	N-Glycan biosynthesis	4.71E-03
ko00565	Ether lipid metabolism	5.38E-03
ko00290	Valine, leucine and isoleucine biosynthesis	5.61E-03
ko00660	C5-Branched dibasic acid metabolism	6.86E-03
ko00100	Steroid biosynthesis	6.90E-03
ko00790	Folate biosynthesis	1.01E-02
ko00010	Glycolysis / Gluconeogenesis	1.20E-02
ko00630	Glyoxylate and dicarboxylate metabolism	1.43E-02
ko00260	Glycine, serine and threonine metabolism	1.44E-02
ko00590	Arachidonic acid metabolism	1.51E-02
ko00062	Fatty acid elongation	1.57E-02
ko04141	Protein processing in endoplasmic reticulum	1.85E-02
ko01040	Biosynthesis of unsaturated fatty acids	1.85E-02
ko00270	Cysteine and methionine metabolism	1.93E-02
ko00950	Isoquinoline alkaloid biosynthesis	2.12E-02
ko00561	Glycerolipid metabolism	2.38E-02

ko00740	Riboflavin metabolism	2.42E-02
ko00910	Nitrogen metabolism	2.45E-02
ko03018	RNA degradation	2.47E-02
ko00710	Carbon fixation in photosynthetic organisms	3.36E-02
ko00402	Benzoxazinoid biosynthesis	3.56E-02
ko04016	MAPK signaling pathway - plant	4.10E-02
ko00250	Alanine, aspartate and glutamate metabolism	4.21E-02
ko00440	Phosphonate and phosphinate metabolism	4.67E-02
ko04122	Sulfur relay system	4.79E-02
ko00592	alpha-Linolenic acid metabolism	4.91E-02
ko00920	Sulfur metabolism	4.92E-02
ko00280	Valine, leucine and isoleucine degradation	5.92E-02
ko00905	Brassinosteroid biosynthesis	6.43E-02
ko00563	Glycosylphosphatidylinositol(GPI)-anchor biosynthesis	6.74E-02
ko00780	Biotin metabolism	7.42E-02
ko03050	Proteasome	9.44E-02
ko00906	Carotenoid biosynthesis	9.64E-02
ko00640	Propanoate metabolism	1.06E-01
ko00531	Glycosaminoglycan degradation	1.09E-01
ko00051	Fructose and mannose metabolism	1.11E-01
ko00750	Vitamin B6 metabolism	1.12E-01
ko00430	Taurine and hypotaurine metabolism	1.34E-01
ko00650	Butanoate metabolism	1.36E-01
ko00960	Tropane, piperidine and pyridine alkaloid biosynthesis	1.41E-01
ko00330	Arginine and proline metabolism	1.47E-01
ko03450	Non-homologous end-joining	1.50E-01
ko00670	One carbon pool by folate	1.61E-01
ko00591	Linoleic acid metabolism	1.62E-01
ko00072	Synthesis and degradation of ketone bodies	1.69E-01
ko00604	Glycosphingolipid biosynthesis - ganglio series	1.73E-01
ko02010	ABC transporters	1.82E-01
ko00020	Citrate cycle (TCA cycle)	2.07E-01
ko00514	Other types of O-glycan biosynthesis	2.46E-01
ko00405	Phenazine biosynthesis	2.54E-01
ko00902	Monoterpenoid biosynthesis	2.57E-01
ko00196	Photosynthesis - antenna proteins	2.59E-01
ko00620	Pyruvate metabolism	2.86E-01
ko03022	Basal transcription factors	3.03E-01
ko04144	Endocytosis	3.09E-01
ko03040	Spliceosome	3.42E-01
ko00480	Glutathione metabolism	3.46E-01
ko00190	Oxidative phosphorylation	3.46E-01
ko00030	Pentose phosphate pathway	3.64E-01
ko00333	Prodigiosin biosyntheses	4.52E-01

ko00310	Lysine degradation	4.60E-01
ko00380	Tryptophan metabolism	4.73E-01
ko01502	Vancomycin resistance	4.91E-01
ko00254	Aflatoxin biosynthesis	4.91E-01
ko00603	Glycosphingolipid biosynthesis - globo and isoglobo series	5.18E-01
ko00073	Cutin, suberine and wax biosynthesis	5.41E-01
ko00261	Monobactam biosynthesis	5.68E-01
ko00908	Zeatin biosynthesis	5.74E-01
ko00195	Photosynthesis	5.87E-01
ko04626	Plant-pathogen interaction	6.34E-01
ko00471	D-Glutamine and D-glutamate metabolism	6.53E-01
ko00523	Polyketide sugar unit biosynthesis	7.01E-01
ko00121	Secondary bile acid biosynthesis	7.01E-01
ko00942	Anthocyanin biosynthesis	7.24E-01
ko01051	Biosynthesis of ansamycins	7.85E-01
ko01053	Biosynthesis of siderophore group nonribosomal peptides	7.85E-01
ko00410	beta-Alanine metabolism	7.85E-01
ko00524	Neomycin, kanamycin and gentamicin biosynthesis	8.07E-01
ko00965	Betalain biosynthesis	8.27E-01
ko00232	Caffeine metabolism	8.51E-01
ko00970	Aminoacyl-tRNA biosynthesis	8.93E-01
ko00785	Lipoic acid metabolism	9.02E-01
ko04120	Ubiquitin mediated proteolysis	9.09E-01
ko00515	Mannose type O-glycan biosyntheis	9.10E-01
ko00943	Isoflavonoid biosynthesis	9.23E-01
ko03013	RNA transport	9.28E-01
ko00601	Glycosphingolipid biosynthesis - lacto and neolacto series	9.30E-01
ko00600	Sphingolipid metabolism	9.56E-01
ko00450	Selenocompound metabolism	9.68E-01
ko00944	Flavone and flavonol biosynthesis	9.79E-01
ko00903	Limonene and pinene degradation	9.94E-01
ko00901	Indole alkaloid biosynthesis	9.95E-01
ko00340	Histidine metabolism	9.99E-01
ko00300	Lysine biosynthesis	9.99E-01
ko03410	Base excision repair	1.00E+00
ko00904	Diterpenoid biosynthesis	1.00E+00
ko00360	Phenylalanine metabolism	1.00E+00
ko00511	Other glycan degradation	1.00E+00
ko00966	Glucosinolate biosynthesis	1.00E+00
ko00941	Flavonoid biosynthesis	1.00E+00
ko00909	Sesquiterpenoid and triterpenoid biosynthesis	1.00E+00
ko00230	Purine metabolism	1.00E+00
ko00500	Starch and sucrose metabolism	1.00E+00
ko00052	Galactose metabolism	1.00E+00

ko00053	Ascorbate and aldarate metabolism	1.00E+00
ko00460	Cyanoamino acid metabolism	1.00E+00
ko03030	DNA replication	1.00E+00
ko03430	Mismatch repair	1.00E+00
ko03440	Homologous recombination	1.00E+00
ko00945	Stilbenoid, diarylheptanoid and gingerol biosynthesis	1.00E+00
ko00940	Phenylpropanoid biosynthesis	1.00E+00
ko03020	RNA polymerase	1.00E+00
ko00520	Amino sugar and nucleotide sugar metabolism	1.00E+00
ko00040	Pentose and glucuronate interconversions	1.00E+00
ko03420	Nucleotide excision repair	1.00E+00
ko00240	Pyrimidine metabolism	1.00E+00
